# Interpersonal trauma moderates the relationship between personality factors and suicidality of individuals with posttraumatic stress disorder

**DOI:** 10.1371/journal.pone.0191198

**Published:** 2018-01-12

**Authors:** Yongjoon Yoo, Hyeon-Ju Park, Soowon Park, Maeng Je Cho, Seong-Jin Cho, Ji Yeon Lee, Soo-Hee Choi, Jun-Young Lee

**Affiliations:** 1 Seoul National University College of Medicine, Seoul, Republic of Korea; 2 Department of Psychiatry, SMG-SNU Boramae Medical Center, Seoul, Republic of Korea; 3 Department of Education, Sejong University, Seoul, Republic of Korea; 4 Department of Psychiatry, Seoul National University College of Medicine, Seoul, Republic of Korea; 5 Department of Psychiatry, Gachon Medical School, Incheon, Republic of Korea; 6 Department of Psychiatry and Neuroscience Research Institute, Seoul National University College of Medicine, Seoul, Republic of Korea; Yale University, UNITED STATES

## Abstract

Individuals with posttraumatic stress disorder (PTSD) are more prone to suicidal ideation and behavior. While those who have experienced interpersonal trauma exhibit more suicidality than those who have experienced non-interpersonal trauma, it is unclear how the traumatic effects are related to an individual’s personality characteristics. This study examined the association between interpersonal trauma and personality factors with suicidality, and elucidated the moderating role of interpersonal trauma in individuals with PTSD. The study included 6,022 participants from the Korean Epidemiologic Catchment Area Study 2011. The Korean Version of Composite International Diagnostic Interview was used for the survey, including the participants’ history of suicidality, the traumas they have experienced, and their PTSD symptoms. The 11-item version of the Big Five Inventory (BFI-11) was used to assess the participants’ personality factors. 76 individuals were diagnosed with PTSD, while 810 had been exposed to trauma but were not diagnosed with any *DSM-IV* mental disorder. Among the individuals with PTSD, those who had experienced interpersonal trauma were more likely to have suicidal ideation than those who had experienced non-interpersonal trauma (*p* = .020; odds ratio [OR] = 3.643; 95% confidence interval of OR = [1.226, 10.825]). High agreeableness and conscientiousness predicted less suicidality in those exposed to non-interpersonal trauma, while predicting *more* suicidality in those exposed to interpersonal trauma. Clinicians examining individuals with PTSD should pay closer attention to the trauma that they have experienced, as well as their personality factors, to provide appropriate treatment.

## Introduction

Posttraumatic stress disorder (PTSD) is a psychiatric disorder that manifests itself after exposure to a potentially traumatic event [1]. Individuals with PTSD actively avoid the triggering traumatic experience(s) and suffer chronic psychological distress *via* intrusive, flashback memories [2]. The presence of PTSD is a well-known risk factor for suicidal ideation and behavior [1,3–8]. Many studies have sought to identify the factors that predict suicide in individuals with PTSD. Some have found an association between PTSD symptoms and suicidality in general populations [7,9–11]. Comorbidities, such as depression, substance use disorder, and psychosis, also predict suicidality in individuals with PTSD [7,8]. However, other predictors of suicidality in PTSD are much less explored.

One possible candidate predictor of suicidality in PTSD is personality. Several personality factors have been associated with suicidality in the general population. Among many different models of personality, the Five Factor Model (FFM) is one of the most widely used that divides the realm of human personality into five dimensions: Openness to experience, conscientiousness, extraversion, agreeableness, and neuroticism [12,13]. A recent systematic review reported that neuroticism and low extraversion, two of the five personality factors defined by the FFM, are associated with suicidal ideation, attempt, and completion [14]. Other personality factors associated with suicidality include hopelessness, impulsivity, and perfectionism [14,15]. Another systematic review revealed that the development of PTSD is positively related to neuroticism, negative emotionality, harm avoidance, novelty-seeking and self-transcendence, and that PTSD symptoms are negatively associated with extraversion, conscientiousness, and self-directedness [16]. However, little progress has been made into which personality factors predict suicidality in individuals with PTSD, and only a couple of studies have considered this topic. One study found that individuals with PTSD who cope with the trauma by suppression are more at risk for suicide [17], while another study discovered a positive relationship between impulsivity and suicide risk in individuals with PTSD [18].

It may also be possible that interpersonal trauma predicts suicidality in individuals with PTSD. Trauma can be classified as either non-interpersonal or interpersonal trauma [19]. The Diagnostic and Statistical Manual of Mental Disorders, Fifth Edition (*DSM-5*) notes that PTSD “may be especially severe or long-lasting when the stressor is interpersonal and intentional” [1]. A number of studies have consistently shown that interpersonal trauma is more likely to cause PTSD than non-interpersonal trauma [20–22] and more likely to cause more severe forms of PTSD [23,24]. Another study reported that interpersonal trauma is correlated with the severity of depression, while non-interpersonal trauma is not [25]. Interpersonal trauma increases suicidality in the general population [26–28], but its effect on individuals with PTSD in terms of suicidality has never been systematically compared to that of non-interpersonal trauma.

Previous studies have shown that among individuals who have experienced interpersonal trauma, social support from family, friends and peers was negatively associated with suicidality [29,30] as well as the development [31] and severity of PTSD [32]. Interpersonal function is one of the main aspects of personality [33]. Moreover, a recent study showed that certain personality factors have significant interactions with the level of interpersonal support as predictors of suicidal ideation [34]. As interpersonal trauma is known to lower interpersonal support and lead to social isolation [35,36], it is essential to consider the effects of interpersonal trauma when examining the relationship between personality and suicidality among individuals with PTSD.

This study investigated the possible predictors of suicidality (evaluated in this study in terms of suicidal ideation, planning, and attempt) in individuals with PTSD. The effects of different personality factors on the suicidality of the individuals with PTSD were analyzed. Moreover, the moderating effects that interpersonal trauma has on these relationships were also examined. As no previous study had examined the relationships between personality factors and suicidality in those who have experienced interpersonal trauma, exploratory hypotheses were chosen. The hypotheses of this study were: (1) Interpersonal trauma predicts suicidal ideation, planning, and attempt in individuals with PTSD compared to individuals who had experienced trauma but were not diagnosed with any mental disorder; (2) Interpersonal trauma moderates the relationship between personality factors and suicidality in the study sample.

## Materials and methods

### Participants and data collection

The research was conducted in compliance with the ethical standards put forth by the Helsinki Declaration. All participants were fully informed about the aims and methods of the study, and written informed consent was obtained prior to participation. The ethics review board of Boramae Medical Center of South Korea approved this study protocol.

The Korean Epidemiologic Catchment Area Study 2011 (KECA-2011) is the third project conducted by the Ministry of Health and Welfare after 2001 and 2006 to estimate the current status of major psychiatric disorders among the general Korean adult population [37]. A total of 246 sampling units from 61 subdivisions were extracted from the 12 catchment areas, encompassing the entire nation, using the multi-stage cluster sampling method based on the 2010 Population Census Data acquired from the National Statistical Office of the Government of the Republic of Korea [38]. At least one household was selected from every sampling unit in a total of 14,204 households; one individual per household was randomly chosen as the respondent. After preliminary surveys by trained field workers, and excluding those who did not meet the study criteria, 6,022 adults, aged 18–74 years, were surveyed *via* in-person interviews from 19 July 2011 to 16 November 2011. The surveys were conducted by 78 interviewers recruited from each catchment area who were trained according to standard protocols developed by the WHO. After excluding those for whom data were either missing or incorrectly entered, 5,909 surveys were analyzed. For the purpose of this study, only the individuals who were diagnosed with PTSD and those who had been exposed to at least one traumatic event but were not diagnosed with any *DSM-IV* mental disorder were included in the sample.

### Diagnostic assessment and measurements

This study used the Korean version of the Composite International Diagnostic Interview (K-CIDI) [39], which is a completely structured diagnostic tool used worldwide. In this study, the PTSD Section of the K-CIDI was used to evaluate the participants’ exposure to previous trauma. The participants were asked whether they had experienced any of the 11 traumas (including ones that could not be classified into any of the 10 listed categories) to assess whether they satisfied Criterion A for the diagnosis of PTSD as described in *DSM-IV-TR* [40]. For the purpose of this study, the 11 trauma categories were divided into two groups. (1) The non-interpersonal trauma (NIT) group included exposure to warfare, life-threatening accident, natural disaster, witnessing serious accidents or death, other stressful accidents and indirect exposure to a stressful accident. (2) The interpersonal trauma (IT) group included those who had been exposed to sexual assault (i.e., unwanted sexual intercourse either by force or under threat), sexual harassment (i.e., having one’s genitals touched or fondled without consent), physical assault, threatened assault with a weapon, incarceration as a prisoner of war, kidnapping, torture, and terrorism. Forbes *et al*. (2014) was referred to for this categorization of trauma [23]. If a participant had experienced more than a single traumatic event, they were asked to choose among them the one that had left the most serious impact and to answer the K-CIDI questions accordingly.

The participants were then evaluated on the other PTSD criteria (B to F) as well. Criterion B describes five re-experience symptoms (repeated unwanted memories, nightmares, flashbacks, psychological distress, and physiological reactivity), among which, one or more are needed to meet the criterion. Criterion C specifies seven avoidance symptoms (avoiding thoughts about the event, avoiding people who are reminders of the event; amnesia, diminished interest, detachment from others, restricted affect, and sense of foreshortened future), among which, three or more are required. Criterion D outlines five hyperarousal symptoms (sleep difficulty, irritability, difficulty concentrating, hypervigilance, and an exaggerated startle response), among which two or more are required. The symptoms as delineated in Criteria B–D should have persisted for more than 1 month (Criterion E) and should cause significant impairment in social, occupational, or other important areas of functioning (Criterion F) for the diagnosis. Those who satisfied all PTSD criteria were diagnosed with PTSD.

The Suicidality Section of the K-CIDI was used to investigate the participants’ history of suicidal ideation, planning, and attempt. The questions were based on those used in the WHO Suicide Prevention Multisite Intervention Study on Suicidal Behaviors [41]. Participants were asked the questions: “Have you ever seriously thought about committing suicide?”, “Have you ever concretely planned suicide?”, and “Have you ever attempted suicide?” to evaluate their lifetime suicidal ideation, planning, and attempts. The participants were asked to answer these questions with either “yes” or “no”, and were assessed as having a history of suicidal ideation, planning, or attempts if they answered “yes” to the respective questions. Suicidality was defined as either suicidal ideation, planning, or attempt for the purpose of this study. The participants were also asked their age at the time of the first suicidal ideation, planning, and attempt.

The FFM is a model of human personality that quantifies personality characteristics in five dimensions (openness to experience, conscientiousness, extraversion, agreeableness and neuroticism). Instruments for the FFM include the Revised NEO Personality Inventory [12] and the 44-item Big Five Inventory (BFI-44) [13]. The shorter, 11-item version of the BFI (BFI-11) has also been developed [42], with two items for each personality factor, except agreeableness, which has three. Each item is scored on a 5-point Likert scale, from “strongly disagree” to “strongly agree”. The score for each personality factor was determined by adding up either the points of each item or, in case of reverse-scored items, 6 minus the point of each item for the personality factor. Items for extraversion are numbered 1R and 6; agreeableness, 2, 7R and 11; conscientiousness, 3R and 8; neuroticism, 4R and 9; and openness, 5R and 10 (R means the item is reverse-scored). The Korean version of the BFI-11 [43] has been included as a part of the Addendum of the K-CIDI survey and was administered to the participants.

### Statistical analysis

SPSS for Windows 18.0 (SPSS Inc., Chicago, IL, USA) was used for the data analysis. Demographic characteristics and the frequencies and percentages of the number of traumas that had been experienced by each participant group were assessed by *t*-tests and chi-square tests. Analysis of covariance was conducted to compare suicidal ideation, planning, and attempts, and the BFI-11 scores between individuals diagnosed with PTSD and those who had been exposed to trauma but did not have any psychiatric disorders with age, sex, and years of education as covariates. These factors were included as covariates because age and sex are known risk factors for suicide [44] whereas age, sex, and the number of years of education are related to the severity of PTSD [45].

Binomial logistic regression was conducted to determine if interpersonal trauma significantly predicted either suicidal ideation, planning, or attempt among individuals with PTSD. Binomial logistic regression was performed to investigate whether BFI-11 scores significantly predicted either suicidal ideation, planning, or attempt among individuals diagnosed with PTSD. PROCESS, a SPSS macro developed by Andrew F. Hayes [46], was employed for these logistic regressions, as well as for analyzing the moderating effects of interpersonal trauma on the relationship between each independent variable and either suicidal ideation, planning, or attempt, with age, sex, and years of education as covariates. The significance level for all statistical tests was set to a two-tailed *p*-value of .05.

## Results

### Demographic characteristics, suicidality, and personality factors of trauma-exposed participants

Among the 5,909 adults who were analyzed for the study, 4,517 had never been exposed to trauma during their lifetime, while 1,480 had experienced at least one traumatic event. Among them, 870 were not diagnosed with any DSM-IV-TR disorder and were classified as the trauma-exposed healthy control (TEHC) group. Yet another 104 were diagnosed with PTSD. Among these, 52 individuals from the TEHC group and 26 from the PTSD group were excluded because they had already had a history of either suicidal ideation, planning or attempt before experiencing the trauma and it was deemed that the effect of trauma on their suicidality may not be able to be properly assessed, as the current study was retrospective in nature. Another 8 individuals from the TEHC group and 2 from the PTSD group were excluded because the traumas they had experienced were either both interpersonal and non-interpersonal or ambiguous in classification. The remaining 76 participants in the PTSD group were exposed to at least a single trauma, and all of them had experienced symptoms necessary for the diagnosis of PTSD as per the *DSM-IV-TR*, including re-experience, avoidance, and hyperarousal.

Table 1 shows the frequencies of the participants who had chosen a particular trauma that produced the worst reactions. No significant difference was observed between the NIT and IT groups in terms of the number of traumatic events they had experienced (*t* = 0.877, *p* > .05). The participants were diagnosed with PTSD during any point of their lifetime; the individuals in the NIT group reported that they had experienced trauma, at an average of 14.5 years ago, while those in the IT group had experienced it an average of 19.0 years ago (*t* = 1.396, *p* > .05; range, 0–60 years).

**Table 1:** Classification of traumatic events and the number of individuals with PTSD and trauma-exposed healthy controls reporting each event as a trauma producing the worst reactions.

	PTSD (*N* = 76)		TEHC (*N* = 810)	
Traumatic events	*N*	%	*N*	%
***Non-interpersonal trauma***	**46**	**100**	**744**	**100**
Warfare	0	0.0	125	16.8
Life-threatening accident	10	21.7	294	39.5
Natural disaster	1	2.2	84	11.3
Witnessing of serious accidents or death	10	21.7	138	18.5
Other stressful accidents	20	43.5	65	8.7
Indirect exposure to stressful accidents	5	10.9	38	5.1
***Interpersonal trauma***	**30**	**100**	**66**	**100**
Sexual assault	6	20.0	4	6.1
Sexual harassment	6	20.0	20	30.3
Physical assault	15	50.0	29	43.9
Threatened assault with a weapon, incarceration as a prisoner of war or kidnapping	2	6.7	13	19.7
Torture or terrorism	1	3.3	0	0.0

Abbreviations: TEHC, trauma-exposed healthy control; PTSD, posttraumatic stress disorder.

Table 2 shows that the 76 individuals in the PTSD group were significantly younger and had a higher percentage of females compared to those in the TEHC group. No significant difference was observed between the two groups in terms of years of education. Individuals in the TEHC group reported that they had experienced an average of 1.4 (*SE* = 0.7) traumatic events, while those in the PTSD group reported experiencing a significantly higher average of 2.2 (*SE* = 1.3) traumatic events (*t* = 5.363; *p* < .001). Among the participants diagnosed with PTSD, 46 belonged to the NIT group, while 30 belonged to the IT group. Among the participants in the NIT group, 28 (60.9%) were female, whereas 26 (86.7%) were female in the IT group, exhibiting a significantly higher ratio than that of the NIT group (*χ*^*2*^ = 5.875, *p* = .015). The mean age of the IT group (43.4 years) was significantly lower than that of the NIT group (51.4 years; *t* = 2.417, *p* = .018). No difference was observed between the two groups in terms of years of education.

**Table 2:** Demographics and suicidality of trauma-exposed participants.

Variables	PTSD (*N* = 76)					TEHC (*N* = 810)	*t* or *χ*^*2*^	*p*
	NIT (*n* = 46)	IT (*n* = 30)	*t* or *χ*^*2*^	*p*	Total (*N* = 76)			
Age, year	51.4 (13.9)	43.4 (14.3)	2.417	.018	48.3 (14.5)	53.8 (15.4)	-2.984	.003
Education, year	10.4 (4.9)	10.8 (5.0)	-0.305	.761	10.6 (4.9)	10.5 (4.8)	0.149	.881
Male, number (%)	18 (39.1)	4 (13.3)	5.875	.015	22 (28.9)	344 (42.5)	5.240	.022
Suicidality								
Suicidal ideation, number (%)	20 (38.5)	21 (70.0)	6.009	.014	41 (53.9)	99 (12.2)	92.542	< .001
Suicidal planning, number (%)	7 (15.2)	8 (26.7)	1.389	.239	15 (19.7)	15 (1.9)	68.635	< .001
Suicidal attempt, number (%)	6 (13.0)	8 (26.7)	2.242	.134	14 (18.4)	17 (2.1)	54.658	< .001

Data are given as mean (SD) for age and years of education, and number (%) for male gender and suicidal ideation, planning and attempt.

Abbreviations: IT, interpersonal trauma; NIT, non-interpersonal trauma; TEHC, trauma-exposed healthy control; PTSD, posttraumatic stress disorder.

Logistic regression was conducted with suicidality as the dependent variable and the diagnosis of PTSD, age, sex, and years of education as independent variables. Individuals in the PTSD group were 7.851 times more likely to have suicidal ideation (95% confidence interval [CI] = [4.725, 13.045]; *p* < .001), 12.507 times more likely to plan for suicide (95% CI = [5.671, 27.580]; *p* < .001) and 9.776 times more likely to attempt suicide (95% CI = [4.512, 21.182]; *p* < .001) compared to the TEHC group.

The BFI-11 scores were compared between the PTSD and TEHC groups. The PTSD group had a significantly lower extraversion (*F* = 6.78; *p* = .009) score but higher neuroticism (*F* = 9.40; *p* = .002) and openness (*F* = 7.85; *p* = .005) scores compared to those in the TEHC group. The two groups were not significantly different in terms of the agreeableness and conscientiousness scores. No significant differences were observed in any of the five dimensions of the BFI-11 score between the NIT and IT groups within the PTSD group.

### Interpersonal trauma as a predictor of suicidality among individuals with PTSD

A logistic regression was conducted for individuals with PTSD, with suicidality as the dependent variable and type of trauma, age, sex, and years of education as independent variables to investigate the effect of interpersonal trauma on suicidality. Individuals in the IT group were 3.643 times more likely to have suicidal ideation (95% CI = [1.226, 10.825]; *p* = .020) compared to those in the NIT group. On the other hand, the type of trauma did not predict any significant change in suicidal planning or attempt.

### Personality factors as predictors of suicidality among individuals with PTSD

Logistic regressions were conducted to determine if BFI-11 scores predicted suicidality in individuals with PTSD and to identify whether the type of trauma had a moderating effect on such relationships. A total of three regressions were performed; each logistic regression was performed with either suicidal ideation, planning, or attempt as the dependent variable and the scores of the five personality factors, the type of trauma, interactions between the type of trauma and the five personality factor scores, age, sex and years of education as independent variables. Among the five personality factors, conscientiousness predicted lower suicidal ideation (odds ratio [OR] = 0.454; 95% CI = [0.163, 0.974]; *p* = .044) and attempt (OR = 0.070; 95% CI = [0.011, 0.459]; *p* = .006). Agreeableness predicted lower suicidal attempt (OR = 0.131; 95% CI = [0.031, 0.562]; *p* = .003) and had a tendency of predicting suicidal ideation (OR = 0.454; 95% CI = [0.201, 1.027]; *p* = .058). Lastly, openness to experience predicted lower suicidal attempt (OR = 0.193; 95% CI = [0.039, 0.951]; *p* = .043). Moreover, significant interactions were found between the type of trauma and the conscientiousness score for suicidal ideation and attempt, between the type of trauma and agreeableness score for suicidal ideation and attempt and between the type of trauma and openness score for suicidal attempt in the PTSD group (Table 3).

**Table 3:** The relationships between BFI-11 personality factor scores and suicidality according to the type of trauma experienced among individuals with PTSD^a^.

	Interaction		Non-interpersonal trauma					Interpersonal trauma				
	*β*	*p*	*β*	SE	*Z*	*p*	OR [95% CI]	*β*	SE	*Z*	*p*	OR [95% CI]
***Suicidal ideation***												
Conscientiousness	0.6523	.033*	-0.269	0.205	-1.315	.189	0.764 [0.511, 1.141]	0.383	0.250	1.534	.125	1.467 [0.899, 2.393]
Agreeableness	0.5467	.044*	-0.243	0.191	-1.272	.203	0.784 [0.540, 1.140]	0.304	0.215	1.416	.157	1.355 [0.890, 2.064]
Extraversion	0.4185	.121										
Neuroticism	0.2523	.746										
Openness	0.0480	.888										
***Suicidal planning***												
Conscientiousness	0.8176	.076	-0.118	0.350	-0.337	.737	0.889 [0.448, 1.765]	0.700	0.335	2.093	.036*	2.014 [1.045, 3.878]
Agreeableness	0.4677	.220										
Extraversion	-0.0433	.894										
Neuroticism	-0.3116	.437										
Openness	0.8936	.101										
***Suicidal attempt***												
Conscientiousness	1.5972	.004**	-1.063	0.437	-2.433	.015*	0.345 [0.147, 0.813]	0.534	0.296	1.806	.071	1.706 [0.956, 3.045]
Agreeableness	1.4101	.003**	-0.623	0.332	-1.876	.061	0.537 [0.280, 1.028]	0.788	0.325	2.425	.015*	2.198 [1.163, 4.155]
Extraversion	-0.2191	.470										
Neuroticism	-0.2491	.475										
Openness	1.0419	.046*	-0.605	0.340	-1.780	.075	0.546 [0.280, 1.063]	0.437	0.336	1.299	.194	1.547 [0.801, 2.990]

Abbreviations: BFI-11, Big Five Inventory, 11-item version; OR, odds ratio; CI, confidence interval; PTSD, posttraumatic stress disorder.

^a^The interaction between the personality factors and interpersonal trauma was investigated, and when the interaction was significant conditional effects of the personality factor were examined for non-interpersonal trauma and interpersonal trauma.

*p < .05

**p < .01.

**Fig 1:**
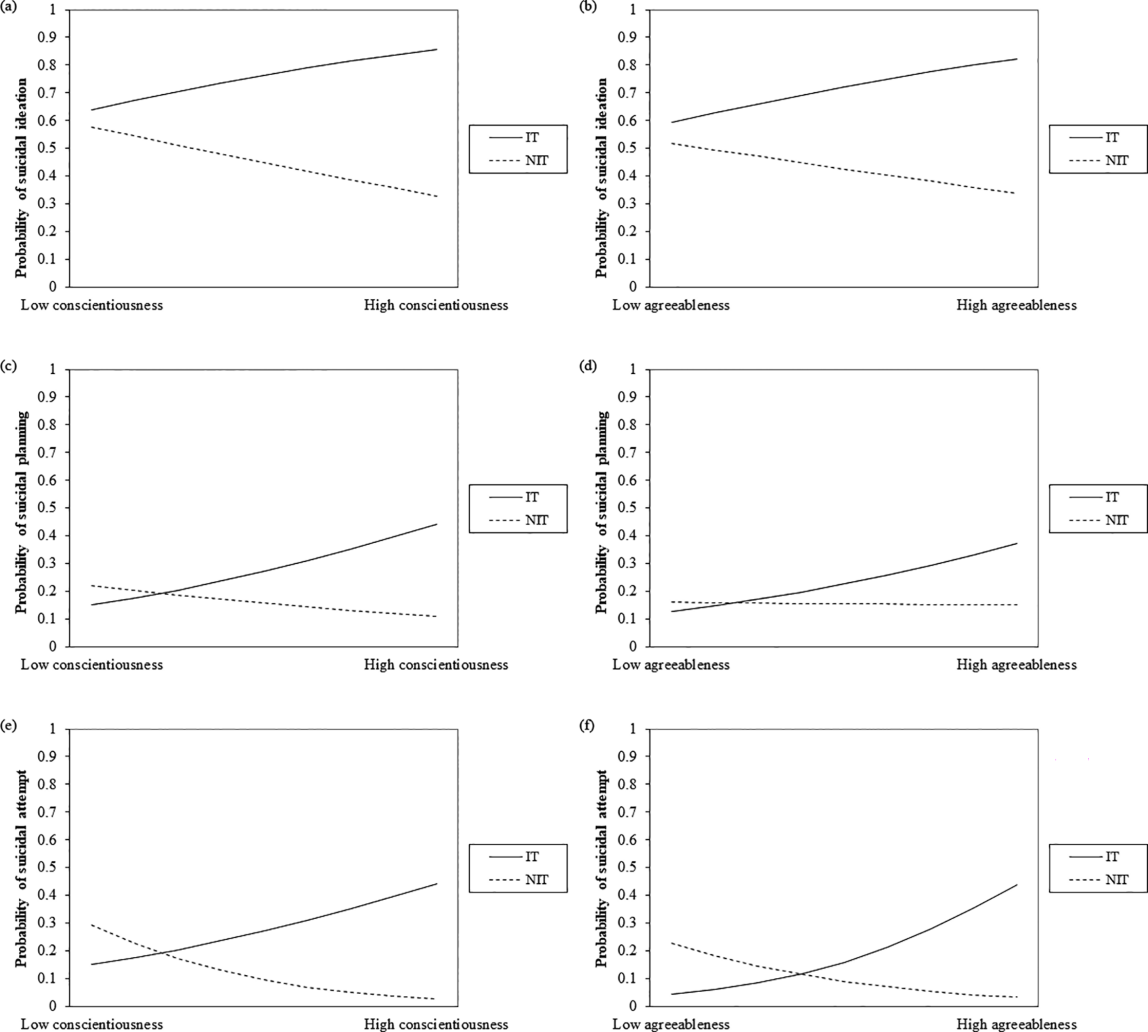
The relationships between BFI-11 personality factor scores and suicidality according to the type of trauma experienced among individuals with PTSD. The effect of the conscientiousness (a) and agreeableness (b) scores on suicidal ideation. The effect of the conscientiousness (c) and agreeableness (d) scores on suicidal planning. The effect of the conscientiousness (e) and agreeableness (f) scores on suicidal attempt. Interaction terms between the respective personality factor scores and the type of trauma were significant for suicidal ideation and attempt. Abbreviations: IT, interpersonal trauma; NIT, non-interpersonal trauma; BFI-11, Big Five Inventory, 11-item version; PTSD, posttraumatic stress disorder.

Interestingly, when the conditional effects of each personality factor score in the NIT and IT groups were examined separately, two completely diametric trends were discovered (Table 3, Fig 1). When the conscientiousness score increased, the likelihood of a suicide attempt in the NIT group decreased (OR = 0.345), whereas that of the IT group had an increasing trend (OR = 1.706; *p* = .071). Similarly, when the agreeableness score increased, the likelihood of a suicide attempt in the NIT group had a decreasing trend (OR = 0.332; *p* = .061), whereas that of the IT group increased (OR = 2.425). The interaction between the openness score and the type of trauma was also significant.

This kind of interaction was much less significant for suicidal ideation and planning. A higher conscientiousness score predicted more suicidal planning in the IT group (OR = 2.014); no significant conditional effect was found in the NIT group. No other significant conditional effect was found for any of the personality factors for either suicidal ideation or planning.

## Discussion

The present findings reveal that interpersonal trauma is a predictor of suicidal ideation in individuals with PTSD. Furthermore, whether the traumatic events experienced by the individuals were interpersonal had moderating effects on the relationships between suicidality and the personality factors. Among the BFI personality factors, conscientiousness and agreeableness predicted suicidality in individuals with PTSD, but in opposite directions for those who have experienced interpersonal trauma and those who have experienced non-interpersonal trauma. These results suggest that clinicians examining individuals with PTSD should pay closer attention to the trauma they have experienced as well as their personality characteristics.

### Interpersonal trauma and suicidality in individuals with PTSD

The statistical analysis showed that individuals with PTSD who had experienced interpersonal trauma as the most traumatizing event were significantly younger and more likely to be female compared to those who experienced non-interpersonal trauma. This was also true for the TEHC group (results not shown). These results are consistent with previous studies, indicating that younger females are more vulnerable to exposure to interpersonal trauma [27]. Moreover, patients with PTSD in the IT group were more likely to have suicidal ideation than those in the NIT group. These results show that interpersonal trauma is a risk factor for suicidality in patients with PTSD, and physicians in a clinical setting should take heed of the types of trauma these individuals experienced.

### Personality factors and suicidality in individuals with PTSD

High neuroticism and low extraversion have been consistently linked with the diagnosis of PTSD [16], and our data are in accordance with this finding. Moreover, the present data also found a higher openness score in the PTSD group compared to that in the TEHC group. High agreeableness and conscientiousness scores predicted lower suicidality in the PTSD group. High agreeableness generally indicates more robust interpersonal support and thus less suicidality and previous studies have found it to be a protective factor for suicidality [47–49]. Similarly, high conscientiousness has also been indicated as a predictor of lower suicidality [47,49]. It seems appropriate that the same would be found for individuals with PTSD as well. Other personality factors traditionally known to be protective in relation to suicidality, such as high extraversion or low neuroticism, were not significant in the PTSD group, possibly because the PTSD group was already biased towards a personality profile of low extraversion and high neuroticism.

### Interaction between personality factors and interpersonal trauma

When moderation by the type of trauma was considered within the PTSD group, an intriguing pattern emerged: participants in the NIT group with high agreeableness and conscientiousness scores were less likely to have attempted suicide, while those in the IT group with the same personality factors were *more* likely to have attempted suicide (Fig 1). According to Costa & McCrae (1992) [12], each of the five personality domains of the FFM comprise six facets. The score of each personality factor obtained from the BFI-11 correlated well with the factor’s subdivisional facet scores that are rated with the longer 44-item version [42]. Therefore, it can be assumed that higher BFI-11 scores can be interpreted as higher scores on individual facets. Further study that makes use of a more detailed personality scale including facet scores is required to elucidate which facets have the strongest predictive power for suicidality in individuals with PTSD.

Agreeableness and conscientiousness have been associated with effortful control [50]. Effortful control is theorized to first emerge during the developmental process; an infant feels frustrated when the infant’s goal-directed activity is hindered, and the infant gradually learns to self-regulate frustration to achieve their goal. Effortful control is then believed to differentiate into two disparate systems: one that deals with frustration from people (agreeableness) and another that deals with frustration from objects and tasks (conscientiousness). In one study, individuals with high levels of effortful control experienced “cold” interpersonal problems, i.e., they were less affiliated with others and more withdrawn [51]. Other studies have noted that agreeable and conscientious individuals are less likely to exhibit interpersonal aggression [52,53]. The existing literature points out that interpersonal trauma “evokes a feeling of betrayal and undermines trust in primary relationships” [54]. Therefore, it may be possible that individuals who are agreeable and conscientious, or who exhibit high levels of effortful control, become distrustful of people and avoidant of interpersonal relationships after suffering interpersonal trauma but, at the same time, wish for social interaction and are less likely to assume an assertive demeanor in a situation of interpersonal conflict. Thus, they are more prone to communicating interpersonal distress through self-harming behavior. Indeed, one study reported that individuals with high interpersonal sensitivity and low interpersonal aggression were more likely to inflict self-harm on themselves and attempt suicide [55]. Further study regarding interpersonal relationships, effortful control, and suicidality could help elucidate how and why unassertive and interpersonally sensitive individuals may resort to self-harming and suicidal behavior after suffering interpersonal trauma.

It is also noteworthy that agreeableness and conscientiousness are two personality factors that comprise the Character matrix used to analyze FFM scores [56]. Character was defined by Allport (1937) as “personal effort judged from the stand-point of some code” based on social standards; it represents moral or ethical activity [57]. According to the matrix, a high-agreeableness-high-conscientiousness individual is described as “moral, respectful, reverent, polite, considerate, sincere, and understanding” and as “the antithesis of the psychopath” [56]. The finding that individuals with PTSD and these types of personality factors are more likely to think about, plan, and attempt suicide after interpersonal trauma suggests that, in addition to their personality factors, a look into their character and morality may aid in further understanding the reasons behind their high suicidality.

### Limitations of the study

This study was a cross-sectional study and thus does not take into account any potential longitudinal changes the participants may have experienced. In particular, the participants’ personality factors were assessed at the time of the survey, invoking the state/trait problem; the trauma that they had experienced may have had an impact on their personality traits, as suggested by a number of studies [58–61]. Notwithstanding such limitations, clinicians may still benefit from the results of this study by assessing the current personality characteristics of individuals with PTSD after their exposure to trauma to estimate their risk for suicidality. A longitudinal study on certain occupational groups at high risk for developing PTSD (e.g., firefighters and policemen) that assesses an individuals’ personality traits and suicidality through time will help further elucidate the relationship between personality, interpersonal trauma and suicidality in individuals with PTSD.

Due to the epidemiological nature of the data, certain measures used were less detailed at the cost of being representative of the whole nation. The study utilized the short, 11-term version of BFI, which may have led to an under-representation of the effects of one or more personality factors. Social support, which is known to play a key role in the relationship between interpersonal trauma and suicidality [2,17,34,62], was not assessed in the KECA-2011 survey and, thus, could not be accounted for. As victims of interpersonal trauma are more likely to have lower social support [63], the moderating effects of interpersonal trauma may be explained, at least partially, by social support. The epidemiological nature of the data also meant that some moderator analyses suffered from relatively small cell sizes. Survivorship bias may also have influenced the results, as those who have successfully completed suicide would not have been able to participate in the survey.

The rate of exposure to traumatic event in the nationally representative data of this study was 24.5%. This is markedly lower than what is reported from a recent worldwide epidemiologic study [64], which found that about 70% of the total population had been exposed to at least a single trauma. It is possible that the threshold for recognizing an event as ‘traumatic’ is higher in the Korean population. Indeed, numerous literatures have pointed out that Asian cultures exhibit markedly lower prevalences of most mental disorders, including anxiety disorders [65,66]. Another possibility is that stigmatization has led to under-reporting the experiencing of traumatic events.

## Conclusion

Overall, this study has found that interpersonal trauma is a predictor of suicidality in individuals with PTSD. Thus, psychiatrists should take notice of trauma that individuals with PTSD have experienced and assess their suicidal risks accordingly. Moreover, high agreeableness and conscientiousness, two personality factors that act as protective factors of suicidality in general, predicted *higher* suicidality in individuals with PTSD who have experienced interpersonal trauma. This result suggests that clinicians treating individuals with PTSD should heed not only the severity of PTSD but also their personality and character as well as the type of trauma they have experienced, and be aware that an agreeable and conscientious person may, in fact, be more vulnerable to suicide after suffering interpersonal trauma.
